# Treatment practice in the elderly patient with chronic lymphocytic leukemia—analysis of the combined SEER and Medicare database

**DOI:** 10.1007/s00277-014-2048-6

**Published:** 2014-03-18

**Authors:** Sacha Satram-Hoang, Carolina Reyes, Khang Q. Hoang, Faiyaz Momin, Sandra Skettino

**Affiliations:** 1 Q.D. Research, Inc, 8789 Auburn Folsom Road C501, Granite Bay, CA 95746 USA; 2 Genentech, Inc, 1 DNA Way, South San Francisco, CA USA; 3 University of California, San Francisco, San Francisco, CA USA

**Keywords:** Chronic lymphocytic leukemia, Comorbidity, Elderly, Treatment, Survival

## Abstract

The median age at diagnosis of chronic lymphocytic leukemia (CLL) is 72, but patients enrolled in randomized trials are often a decade younger. Therapy selection and outcomes in the older, comorbid population are less understood. We evaluated treatment patterns and outcomes among 2,985 first primary CLL patients from the linked Surveillance, Epidemiology, and End Results–Medicare database. There were 151 chlorambucil (CLB), 594 rituximab monotherapy (R-mono), 696 rituximab + intravenous chemotherapy (R + IV Chemo), and 1,544 IV chemo-only patients. Patients administered CLB and R-mono were the oldest and had the highest comorbidity burden while patients receiving R + IV Chemo were the youngest and had the lowest comorbidity burden (*p* < 0.0001). In the multivariate survival analysis, receipt of R + IV Chemo was associated with significantly lower mortality risk vs. IV Chemo-only (hazard ratio (HR) = 0.73; 95 % confidence interval (CI) 0.62–0.87) and a non-significant mortality risk reduction with R-mono vs. CLB (HR = 0.47; 95 % CI: 0.21-1.05). Older age and increasing comorbidity score were significantly associated with higher mortality. These findings suggest that chemoimmunotherapy is more effective than chemotherapy in an elderly population with a high prevalence of comorbidity, and this extends the conclusions from clinical trials in younger, medically fit patients.

**Electronic supplementary material:**

The online version of this article (doi:10.1007/s00277-014-2048-6) contains supplementary material, which is available to authorized users.

## Introduction

Chronic lymphocytic leukemia (CLL) is a lymphoproliferative disorder that predominantly affects the elderly with a median age at diagnosis of 72 years and almost 70 % of new cases diagnosed in individuals 65 years or older [1, 2]. Elderly patients are often compromised by concurrent pathological conditions and/or organ function decline [3], and 46 % of these newly diagnosed elderly CLL patients have major comorbidities present [4].

Currently, immunochemotherapy with fludarabine, cyclophosphamide, and rituximab (FCR) is considered the standard of care in previously untreated, medically fit patients with CLL and has shown a significant survival benefit in this population [5]. Among older patients who are frail or medically unfit, however, fludarabine-based chemoimmunotherapy is generally less well tolerated [6]. Chemoimmunotherapy is often withheld, or physicians may opt to reduce dosage in order to decrease the risk of occurrence and/or severity of adverse events [7]. A number of other treatment approaches are employed for older patients with comorbidity and/or other age-related organ function decline. These treatments may include chlorambucil (CLB), rituximab monotherapy, fludarabine monotherapy, or bendamustine [8, 9]. There is limited information on these treatment approaches in elderly or frail CLL patients.

In July 2006, Medicare coverage was expanded to include prescription drugs under Medicare Part D. CLB is covered under Medicare Part D, and data for its use are newly available in the linked Surveillance, Epidemiology, and End Results (SEER)–Medicare dataset for the time period of 2007 to 2009. In elderly or medically unfit patients, results from randomized controlled trials as well as real-world data that compare current treatment approaches are lacking. The objectives of this study were to identify patient characteristics associated with receiving different treatment regimens and to evaluate the impact of these treatments on clinical outcomes in a real-world cohort of elderly, demographically diverse CLL patients.

## Methods

### Data sources

Data from the SEER–Medicare linked database were used for these analyses, and institutional review board approval was waived because there are no personal identifiers in the SEER–Medicare database. This database is a collaborative effort between the National Cancer Institute (NCI), the SEER registries, and the Centers for Medicare & Medicaid Services and provides information on Medicare patients included in SEER, a nationally representative collection of 18 population-based registries of all incident cancers from diverse geographic areas [10]. The linked database includes all incident cancer patients reported to the SEER registries and cross-matched with a master file of enrollees in Medicare [11] with approximately 97 % of persons 65 years or older eligible for Medicare. Inpatient care, skilled nursing care, home healthcare, and hospice care are covered services under Medicare Part A while Part B reimburses for physician and outpatient care with about 95 % of beneficiaries subscribing to Part B. The SEER–Medicare linkage includes all Medicare-eligible persons reported to SEER through 2007 and their Medicare claims for Part A (inpatient) and Part B (outpatient and physician services) through 2009.

### Study population

Eligibility criteria for the analysis included a diagnosis of first primary CLL, age 66 years or older, continuous enrollment in both Medicare Part A and B in the 12 months preceding the diagnosis, and receipt of any oral or infused chemotherapy or immunotherapy between 2001 and 2009 (Supplementary Fig. 1). The exclusion criteria included a date of death that occurred prior to or during the same month as the month of diagnosis (*n* = 689) and enrollment in a health maintenance organization (HMO) at any time during the 12 months prior to diagnosis (*n* = 2,714) because treatment and outcome data would not be available.

### Study variables

The SEER program collects data on: patient age, race/ethnicity, residence, and socioeconomic status (income and education per census tract), and primary tumor site, tumor morphology, stage at diagnosis, first course of treatment, and follow-up for vital status. Median annual household income at the census tract level and the percentage of the adult population who completed specific levels of education at the ZIP code level were used as a proxy for socioeconomic status. The SEER site code was used to identify patients diagnosed with CLL. Stage at diagnosis is not available for CLL in the SEER database. A proxy for stage was based on the Binet and Rai et al. [12, 13] staging systems with patients classified as “advanced stage” if anemia and/or thrombocytopenia were present in the claims data [14].

Data were abstracted from five merged SEER–Medicare files to identify claims for chemotherapy or immunotherapy administration [15]. These included the Medicare provider analysis and review (MEDPAR), carrier claims (NCH), outpatient claims (OUTSAF), durable medical equipment (DME), and the prescription drug event (PDE) files; each of these files provides calendar year summaries of reimbursed services.

In July 2006, Medicare coverage was expanded to include prescription drugs under Medicare Part D. Chlorambucil is covered by Medicare Part D, and data for its use were only available from 2007 to 2009 in the PDE claims file. Chemotherapy and immunotherapy were characterized and quantified using the International Classification of Disease (ICD) diagnosis codes, ICD procedural codes, Current Procedural Terminology (CPT) codes, Healthcare Common Procedure Coding System (HCPCS) codes, and revenue center codes. Chemotherapy claims were searched for specific drug codes to identify the type of chemotherapy administered to patients. The absence of these claims was interpreted as evidence of no treatment. The first chemotherapy claim after diagnosis indicated the start of therapy. Patients were classified into one of four treatment groups based on all chemotherapy administered during the first 60 days following initiation of treatment. The four groups included CLB, rituximab monotherapy (R-mono), rituximab and intravenous chemotherapy (R + IV Chemo), and intravenous chemotherapy alone (IV Chemo-only).

The NCI comorbidity index [16] was calculated for each patient using diagnosis and procedure codes in the Medicare Part A and B claims files to identify the 15 non-cancer comorbidities from the Charlson Comorbidity Index [17]. A weight is assigned to each condition based on its potential to influence 2-year mortality, and these weights are summed to obtain an index for each patient. The index accounts for the number and severity of the conditions, with higher scores indicating a greater burden of comorbid disease.

Comorbidity was also examined using the number of involved organs systems in the Cumulative Illness Rating Scale (CIRS) [18, 19]. CIRS assesses patient comorbidity by physicians specifying the presence or absence of pathology in each of the 14 organ systems and the severity of impairment in each involved system. Given that disease severity data were not available in this claims-based analysis to calculate a total CIRS score for each patient, we presented the number of involved CIRS organ systems. The number of involved CIRS organ systems was quantified using diagnosis and procedure codes in the Medicare Parts A and B claims files to identify specific conditions that relate to each organ system category.

The NCI Comorbidity Index and the CIRS are among the most valid and reliable measures of multi-morbidity [20, 21]. For both comorbidity definitions, Medicare claims during the year preceding the diagnosis were evaluated to determine the baseline comorbidity burden for each patient. Specific conditions were required to appear on at least two different claims that were more than 30 days apart to ensure that “rule out” diagnoses were not counted as comorbid conditions.

The date of death was assigned by using the Medicare date or SEER date of death if the Medicare date was missing. All other patients were assumed to be alive at the end of the follow-up period on December 31, 2009, although they may have been censored earlier for other reasons such as the diagnosis of a second primary cancer or lack of availability of Medicare claims data.

### Statistical analysis

All statistical analyses were performed using SAS software, version 9.1.3 (SAS Institute Inc., Cary, NC, USA). Demographic and clinical baseline characteristics among the patients initiating treatment between 2001–2009 (excluding CLB) and 2007–2009 (the period for which CLB data were available) were summarized descriptively. The chi-square test for categorical variables and analysis of variance or *t* tests for continuous variables were performed to determine differences between groups. We considered a *P* value <0.05 to be statistically significant.

The survival analyses compared CLB to R-mono from 2007 to 2009 because this was the time during which Part D Medicare claims data on CLB were available. Patients initiating treatment with R + IV Chemo and IV Chemo-only from 2001 to 2009 were compared. The survival analyses to assess overall risk of death were based on a comparison of two approaches as a sensitivity exercise which included the Cox proportional hazards regression and the propensity score-weighted Cox proportional hazards regression model. The traditionally adjusted Cox proportional hazards regression model allowed us to explore independent predictors of mortality, while the propensity score-weighted model is limited to assessing the effect of treatment. A comparison of these two models yielded almost identical results of treatment effect.

In the Cox proportional hazards regression, we adjusted the model for confounders that were selected from demographic and clinical characteristics using the backward elimination strategy [22]. In the propensity score weighted model, we used multinomial logistic regression to calculate a propensity score, which represents the conditional probability that a patient would receive a specific treatment given each patient’s pretreatment variables such as age, gender, and comorbidities [23]. Follow-up was calculated from the date of treatment initiation until the first occurrence of a censoring event including date of death, development of a second primary tumor, the last date for which Medicare claims were available, or the end of the follow-up period on December 31, 2009*.* Kaplan–Meier survival curves and corresponding log-rank tests examined unadjusted OS by treatment group.

## Results

### Treatment patterns

**Fig 1:**
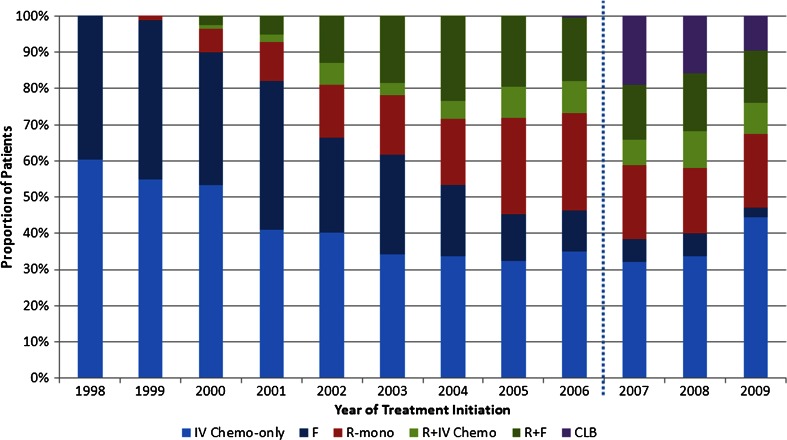
Treatment type by year of initiation. *IV chemo-only* intravenous chemotherapy only, *F* fludarabine containing subset of IV chemo-only, *R-mono* rituximab monotherapy, *R + IV chemo* rituximab plus intravenous chemotherapy, *R + F* fludarabine containing subset of R + IV chemo, *CLB* chlorambucil. Note: Part D chlorambucil data available for 2007–2009 only

Of the patients who met the study eligibility criteria, 594 (20 %) were administered R-mono, 696 (23 %) were treated with R + IV Chemo, 1,544 (52 %) received IV Chemo-only, and 151 (5 %) received CLB in the first-line setting. Treatment with rituximab increased during the study time period (Fig. 1). During the period of availability (2007–2009) of all four treatment groups, the rate of IV Chemo-only use in first-line treatment was 41 %, R + IV Chemo was 24 %, R-mono was 20 %, and oral CLB was 16 %.

Table 1 shows the distribution of the specific types of therapies received in the cohort. Of the 696 patients receiving R + IV Chemo, 495 (71 %) received a regimen including F, and 376 (54 %) received a regimen containing C. There were 192 (28 %) patients identified as receiving both F and C with rituximab and are included in the estimates of the two groups. Of the 1,544 patients receiving IV Chemo-only, 486 (31 %) received a regimen including F, and 167 (11 %) received a regimen containing C. There were 63 (4 %) patients identified as receiving both F and C and are included in the estimates of both groups.

**Table 1:** First-line therapy initiated from 2001 to 2009

Treatment type	Number	Percent
R-mono	594	100
R + IV chemo		
R + CHOP	46	6.6
R + CVP	76	10.9
R + FC	192	27.6
R + F	303	43.5
R + C	62	8.9
R + other	17	2.4
Total	696	100
IV chemo-only		
CHOP	82^a^	5.3
CVP		
FC	63	4.1
F	423	27.4
C	22	1.4
Other chemo	345	22.3
Unknown chemo	609	39.4
Total	1,544	100

*R-mono* rituximab monotherapy; *R + IV chemo* rituximab plus intravenous chemotherapy; *IV chemo-only* intravenous chemotherapy only; *CHOP* cyclophosphamide, doxorubicin, vincristine, and prednisone; *CVP* cyclophosphamide, vincristine, and prednisone; *FC* fludarabine and cyclophosphamide

^a^Cells with counts of less than 11 are combined in compliance with the National Cancer Institute data use agreement for small cell sizes

### Patient characteristics

Table 2 shows patient baseline characteristics among the patients initiating treatment between 2001–2009 (excluding CLB) and 2007–2009 (including CLB). Patients treated with CLB (mean age 77), R-mono (mean age 77), and IV-Chemo only (mean age 76) were older at diagnosis compared to those administered R + IV Chemo (73 years; *P* < 0.0001). However, looking at the oldest age category (>80), almost one third (32 %) of patients treated with R-mono were older than 80, followed by 28 % for CLB, 24 % for IV-Chemo only, and 7 % for R + IV Chemo (*P* < 0.0001). Significantly more patients treated with CLB and R-mono were females compared with the other two treatment groups (*P* < 0.05). Patients receiving R-mono were more likely to have advanced stage disease (59 %) compared to the other treatment groups (45–48 %; *P* < 0.05). R-mono patients also had the highest comorbidity burden while R + IV Chemo patients had the lowest comorbidity burden (*P* < 0.001). Forty-eight percent of R-mono patients had ≥4 organ systems affected by comorbidity, and 44 % had an NCI score ≥1, while 31 % of R + IV Chemo patients had ≥4 organ systems affected by comorbidity and 28 % had an NCI score ≥1. CLB patients were also more likely to be non-white and resided in areas of lower income and educational levels compared to the other three groups. There were similar proportions of patient characteristics for R-mono, R + IV Chemo, and IV Chemo-only when looking at the 2001–2009 cohorts.

**Table 2:** Baseline characteristics for the population initiating therapy during the period 2001–2009 and 2007–2009

	Initiating therapy 2001–2009				Initiating therapy 2007–2009				
	R-mono (*N* = 594)	R + IV chemo (*N* = 696)	IV chemo-only (*N* = 1,544)	*P*	CLB^b^ (*N* = 151)	R-mono (*N* = 186)	R + IV chemo (*N* = 224)	IV chemo-only (*N* = 386)	*P*
	%	%	%		%	%	%	%	
Age at diagnosis				<0.0001					<0.0001
66–70	19.9	35.9	20.8		17.9	24.2	35.3	19.9	
71–75	23.9	28.7	27.3		22.5	23.1	32.6	26.7	
76–80	24.9	25.0	27.7		31.8	21.0	25.0	29.8	
>80	31.3	10.3	24.2		27.8	31.7	7.1	23.6	
Gender				0.0150					0.0313
Male	54.0	61.9	59.1		45.7	48.9	58.5	56.5	
Female	46.0	38.1	40.9		54.3	51.1	41.5	43.5	
Race/ethnicity				0.9726					0.0008
White	92.4	92.2	92.1		83.4	93.5	92.4	93.8	
Non-white	7.4	7.6	7.7		16.6	6.5	7.6	6.2	
Stage^a^				<0.0001					0.0261
Non-advanced	36.9	52.0	47.0		51.7	40.9	54.9	52.3	
Advanced	63.1	48.0	53.0		48.3	59.1	45.1	47.7	
Number of involved CIRS organ systems				<0.0001					0.0292
0	9.4	15.7	12.1		9.3	8.6	10.7	8.3	
1–3	45.3	53.9	51.2		53.0	43.5	58.5	49.5	
≥4	45.3	30.5	36.7		37.7	47.8	30.8	42.2	
NCI comorbidity score				<0.0001					0.0008
0	55.4	69.4	59.5		62.3	54.8	71.9	60.4	
1	25.9	20.8	24.7		19.2	23.1	21.0	23.8	
2	10.9	6.5	9.5		9.3	11.3	6.7	8.8	
≥3	7.7	3.3	6.3		9.3	10.8	0.4	7.0	
Geographic region				0.0059					0.0900
Midwest	12.3	12.4	11.9		7.9	12.9	11.6	8.3	
Northeast	5.4	7.0	6.5		9.9	3.2	7.1	6.5	
South	40.4	44.0	48.6		52.3	43.0	47.8	47.2	
West	41.9	36.6	33.0		29.8	40.9	33.5	38.1	
Median income quartiles				0.0560					
1—low	19.4	23.6	24.9		31.1	17.7	22.8	24.9	0.0959
2	23.6	22.4	24.0		26.5	23.1	22.3	21.0	
3	27.8	24.1	25.5		23.2	28.5	25.9	23.6	
4—high	28.3	28.7	24.4		18.5	30.6	28.1	29.0	
Education									
% Less than high school	16.67	17.33	17.92	0.0695	20.01	16.83	16.73	17.03	0.0272
% High school only	26.55	26.58	27.87	0.0024	29.74	26.36	26.08	26.59	0.9135
% Some college	28.38	28.07	27.62	0.0648	26.34	27.68	27.64	27.95	0.0009
% At least a college degree	28.22	28.03	26.60	0.0597	23.91	29.13	29.55	28.42	0.9387

*CIRS* Cumulative Illness Rating Score, *CLB* chlorambucil, *IV Chemo-only* intravenous chemotherapy only, *NCI* National Cancer Institute, *R + IV Chemo* rituximab plus intravenous chemotherapy, *R-mono* rituximab monotherapy

^a^Advanced stage disease was approximated by the presence of anemia and/or thrombocytopenia in the claims data

^b^Part D chlorambucil data were only available for the 2007 to 2009 time period

### Clinical outcomes

**Fig 2:**
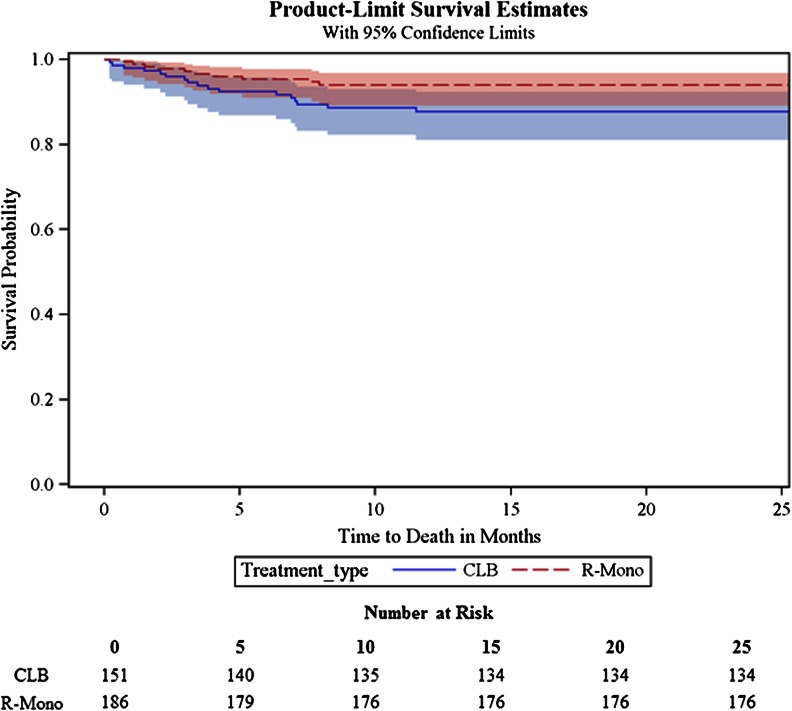
Unadjusted overall survival of CLB vs. R-mono (2007–2009)

The survival analyses compared CLB with R-mono for the time period 2007 to2009 and R + IV Chemo with IV Chemo-only from 2001 to 2009. The unadjusted overall survival was higher for patients administered R-mono compared with CLB (log rank *P* = 0.0478; Fig. 2). Although the median survival was not reached after 1 year of follow-up, the proportion of patients surviving were 95 % [standard error (SE) = 0.79] in the R-mono group and 89 % (SE = 0.88) in the CLB group. The multivariate Cox regression survival analysis (Table 3) revealed a non-significant decrease in mortality risk among patients treated with R-mono compared with CLB patients (HR 0.466; 95 % confidence interval (CI) 0.21–1.05). This finding was confirmed in the propensity weighted Cox regression. The full Cox regression model included age, sex, race, stage, NCI comorbidity score, geographic region, income, and year of diagnosis. The risk estimates were unchanged when replacing NCI comorbidity score with number of involved CIRS organ systems in the model.

**Table 3:** Adjusted overall survival, CLB vs. R-mono (2007–2009)

Covariates	Number	Multivariate Cox regression reduced model^a^		Propensity-weighted Cox regression^b^	
		HR	95 % CI	HR	95 % CI
Treatment					
CLB (ref)	151				
R-Mono	186	0.466	0.21–1.05	0.548	0.27–1.12
Age at diagnosis					
71–75 (ref)	77				
76-80	87	2.932	0.89–9.65		
>80	101	3.410	1.06–10.95		
NCI comorbidity score					
0 (ref)	196				
1	72	2.369	0.83–6.78		
2	35	2.572	0.90–7.32		
≥3	34	3.057	0.94–9.90		

*CI* confidence interval, *CLB* chlorambucil, *HR* hazard ratio, *R-mono* rituximab monotherapy

^a^Reduced model by backward elimination. Full model included age, sex, race, stage, comorbidity score, geographic region, income, and year of diagnosis

^b^Propensity score weighted for age, sex, race, stage, comorbidity score, geographic region, income, and year of diagnosis

^c^Advanced stage disease was approximated by the presence of anemia and/or thrombocytopenia in the claims data

**Fig 3:**
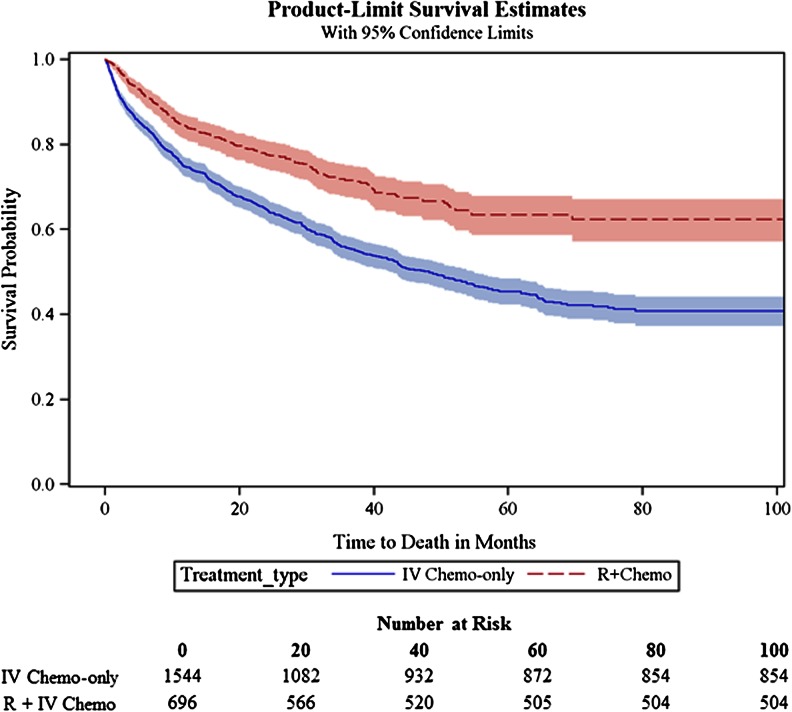
Unadjusted overall survival of IV chemo-only vs. R + IV chemo (2001–2009)

The unadjusted overall survival was significantly higher for R + IV Chemo compared with the IV Chemo-only group (log rank *P* < 0.0001; Fig. 3) with 5-year overall survival rates of 73 % (SE = 1.08) for R + IV Chemo compared with 56 % (SE = 0.94) for IV Chemo-only. The multivariate Cox regression survival analysis adjusted for age, gender, race, stage, comorbidity, income, diagnosis year, and geographic region revealed a 27 % lower risk of death for R + IV Chemo patients compared with IV Chemo-only patients (Table 4). This finding was confirmed in the propensity weighted Cox regression with almost identical rates. Increasing age and increasing NCI comorbidity score were associated with significantly higher risks of death, while female gender and white race had significant protective effects on mortality. The risk estimates were unchanged when replacing NCI comorbidity score with number of involved CIRS organ systems in the model.

**Table 4:** Adjusted overall survival, IV chemo-only vs. R + IV chemo (2001–2009)

Covariates	Number	Multivariate Cox regression reduced model^a^		Propensity-weighted Cox regression^b^	
		HR	95 % CI	HR	95 % CI
Treatment					
IV chemo-only (ref)	1,544				
R + IV chemo	696	0.73	0.62–0.87	0.75	0.64–0.87
Age at diagnosis					
66–70 (ref)	571				
71–75	621	1.25	1.03–1.53		
76–80	602	1.44	1.19–1.76		
>80	446	2.22	1.81–2.71		
Gender					
Male (ref)	1,343				
Female	897	0.81	0.70–0.93		
Race/ethnicity					
Non-white (ref)	172				
White	2,068	0.72	0.57–0.90		
NCI comorbidity score					
0 (ref)	1,401				
1	526	1.10	0.94–1.30		
2	192	1.37	1.09–1.72		
≥3	121	1.76	1.37–2.28		

*CI* confidence interval, *HR* hazard ratio, *IV chemo-only* intravenous chemotherapy only, *NCI* National Cancer Institute, *R + IV chemo* rituximab plus intravenous chemotherapy

^a^Reduced model by backward elimination. Full model included age, sex, race, stage, comorbidity score, geographic region, income, and year of diagnosis

^b^Propensity score weighted for age, sex, race, stage, comorbidity score, geographic region, income, and year of diagnosis

^c^Advanced stage disease was approximated by the presence of anemia and/or thrombocytopenia in the claims data

Several sensitivity analyses were conducted to explore factors affecting the main results. To confirm that the reduction in mortality was significant for specific treatment groups within the broader R + IV Chemo vs. Chemo-only groups, we restricted the primary analysis to patients receiving R–F vs. F-only and R–FC vs. FC. R–F was associated with a 44 % reduction in mortality compared to F-only (HR = 0.56; 95 % CI 0.43–0.73), adjusting for all other covariates in the model. Although results were directionally similar to the primary model with a 29 % reduction in mortality for R–FC compared to FC (HR = 0.71; 95 % CI = 0.44–1.12), the findings were not statistically significant due to the small sample of patients receiving FC (*n* = 63). We also stratified the analyses by different categories of comorbidity burden. In the subpopulation of patients with an NCI comorbidity score of 0, there was a 27 % reduction in mortality with R + IV Chemo compared to Chemo-only (HR = 0.73; 95 % CI = 0.59–0.89). R + IV Chemo was particularly effective among patients with an NCI comorbidity score of 1 (HR = 0.59; 95 % CI 0.41–0.85) with a 41 % reduction in mortality. Although the mortality risks were also lower in patients with an NCI comorbidity score of ≥2 (HR = 0.91; 95 % CI = 0.58–1.46), the sample is small and the confidence interval is wider showing no statistical significant difference.

## Discussion

Results of this retrospective registry-based analysis highlight that the choice of treatment regimens for first-line therapy of CLL varied according to the clinical and demographic characteristics of patients. Notably, patients receiving single-agent rituximab or CLB were the oldest and had the highest comorbidity burden. In contrast, patients receiving R + IV Chemo were the youngest and had the lowest comorbidity burden. This is in line with Current National Comprehensive Cancer Network (NCCN) guidelines that suggest that frail patients or those with significant comorbidity be treated with oral therapy (chlorambucil) ± rituximab or with single-agent rituximab. Chemo-immunotherapy (e.g., FCR) is preferred for patients under 70 without significant comorbidity [24]. Our observations are also consistent with findings from prospective clinical trials indicating that chemoimmunotherapy is associated with the most favorable outcomes for medically fit, relatively younger patients [5, 25] while those who are older or less medically fit may not be able to tolerate the toxicities associated to it [26]. Recently, the German CLL Study Group published their pivotal trial of 781 previously untreated CLL patients (median age of 73) with coexisting conditions [27]. This study demonstrated that rituximab–chlorambucil compared to chlorambucil monotherapy increased response rates and prolonged progression-free survival. Even greater improvements with the novel anti-CD20 agent obinutuzumab (GA101) plus chlorambucil compared to chlorambucil monotherapy were also reported.

The high level of R-mono usage among the very elderly in actual clinical practice is an important finding. Although this is listed as an option on the NCCN guidelines for this age group with comorbidities, there is a scarcity of data on this therapeutic approach [28–30]. Prior to 2007, rituximab may have been used in combination with CLB; however, we cannot confirm this hypothesis since CLB use was not available in the SEER–Medicare dataset at that time.

Patients receiving R-mono were also more likely to be diagnosed with advanced disease. This treatment choice is most likely a consequence of the medical fitness of patients as indicated by the older age at diagnosis and higher comorbidity burden rather than tumor burden per se. Another potential reason for underutilization of IV Chemo-only or R + IV Chemo in patients with advanced disease may be due to co-existent renal disease as fludarabine use is contraindicated in patients with severe renal impairment. This is consistent with the finding that <4 % of IV Chemo-only or R + IV Chemo patients had renal impairment whereas 9 % of patients receiving R-mono and 7 % receiving CLB had renal impairment in our study. Additionally, some clinicians may inappropriately consider marrow failure (manifested by the development or worsening of anemia and/or thrombocytopenia) as an indication for caution and may opt to dose reduce or avoid chemotherapy, rather than treat [31]. It may also be possible that older patients are more reluctant to seek medical attention and therefore are diagnosed at a later stage. Further research is warranted to assess the validity of this hypothesis.

Although our follow-up time was short, there was a non-significant trend for higher unadjusted overall survival rates for patients receiving R-mono (95 %) compared with CLB (89 %) after 1 year of follow-up and a similar observation in the multivariate model which showed a non-significant trend toward decreased mortality risk in R-mono patients. A longer follow-up period would shed more light on this finding. The adjusted multivariate analysis for R + IV Chemo and IV Chemo-only revealed a significantly lower risk of death for R + IV Chemo patients suggesting that chemoimmunotherapy is a more effective treatment regimen even in this cohort of older patients, many of whom had significant comorbidities [5, 25].

The most frequently administered regimen in patients included in this study, those who are 66 years or older with a diagnosis of first primary CLL, was IV Chemo-only followed by rituximab with or without chemotherapy. Notably, the use of rituximab increased during the study interval from 11 % in 2000 to 44 % in 2009. Such an increase was also reported by Danese et al. [14] with a shorter follow-up period through the end of 2007. The higher rituximab treatment rates noted in the current study may be related to the extension of the follow-up period to December 31, 2009 as the evidence for the efficacy of rituximab grew [5, 25, 32–34] and increasing numbers of clinicians opted to treat their patients with rituximab-based regimens. An examination of the period of availability (2007–2009) of all four treatment groups shows that the rate of IV Chemo-only was 41 %, R + IV Chemo was 24 %, R-mono was 20 %, and oral CLB was 16 %.

The variation in practice patterns in this demographically diverse patient population deserves mention. Treatment selection varied by sex, race, income, and educational levels similar to patterns observed in prior oncology research [35, 36]. In our study, patients receiving CLB were more likely female, non-white, and lived in areas of lower socioeconomic status. The more frequent use of CLB among females was perhaps even more notable given that females were less likely to receive any therapy vs. males. Reducing the disparity of nonclinical factors on the receipt of cancer therapy may reduce the adverse impact on outcomes among these patients. Further research is warranted to better quantify the full spectrum of nonclinical factors that contribute to receipt of cancer therapy in order to develop strategies that facilitate appropriate cancer care for all patients.

The SEER–Medicare database offers comprehensive information about inpatient and outpatient claims, covered services, all claims regardless of residence or service area, and longitudinal data with claims for services from the time a person is eligible for Medicare until their death. To our knowledge, this is the first study to include real-world treatment patterns and outcomes for CLB in an elderly population of CLL patients. However, several factors need to be considered in interpreting the findings.

Medicare only began to offer the Part D benefit for pharmacy claims on January 1, 2006. Therefore, it is possible that patients may have received CLB prior to their first claim recorded in the Medicare Part D claims files and our results may underestimate the number of patients who were treated with CLB. In addition, Medicare claims data more accurately identify agents that are intravenously administered since oral agents are covered under Medicare Part D [37], and it is estimated that only 53 % of Medicare beneficiaries with a first primary of any cancer were enrolled in Medicare Part D in 2009 [38]. The low rate of claims for CLB may be due, in part, to missing information for patients who were solely treated with oral agents prior to 2006 that would be covered by Medicare Part D.

The SEER registry does not collect staging information for leukemia, and our surrogate for stage (including claims for anemia and thrombocytopenia as a marker of disease severity) may not adequately assess stage in all patients in our study. Further, the use of anemia as a surrogate for advanced disease may be subject to bias as there are multiple causes of anemia in the elderly patient. Foremost among these is renal impairment which increases significantly in incidence at this age group. However, <5 % of our entire cohort had renal impairment making it unlikely that this factor introduced significant bias into the analysis.

The SEER–Medicare database also does not provide data on performance status or lifestyle factors, which could have influenced clinicians’ decisions regarding specific therapeutic regimens administered to patients included in our analysis. In addition, we did not have information about treatment patterns and predictors for patients enrolled in health maintenance organizations (HMO) or fee-for-service plans since these data are not collected by Medicare. Treatment patterns, prognosis, and complications may differ between these alternative health care plans and Medicare enrollees, and this would be a productive area for additional evaluation.

However, despite these limitations, this study provides new information regarding the treatment patterns and outcomes in an elderly, medically unfit patient population from a large population-based registry that includes a wide geographic representation of patients in the USA. Patients treated with R-mono and CLB were found to be older at diagnosis and had a higher comorbidity burden, while patients treated with R + IV Chemo were the youngest at diagnosis and had the lowest comorbidity burden. Adjusting for these differences in the survival analysis showed a significant mortality risk reduction with R + IV Chemo vs. IV Chemo-only and a non-significant mortality risk reduction with R-mono vs. CLB. These findings suggest that chemoimmunotherapy is more effective than chemotherapy in an elderly population with a high prevalence of comorbidity, and this extends the conclusions from clinical trials in younger, medically fit patients.

## Electronic supplementary material

Below is the link to the electronic supplementary material.

ESM 1 (JPEG 80.1 kb)

ESM 1 (JPEG 39.5 kb)

## References

[1] Howlader N, Noone A, Krapcho M (2011). SEER cancer statistics review, 1975–2009 (vintage 2009 populations).

[2] Siegel R, DeSantis C, Virgo K (2012). Cancer treatment and survivorship statistics, 2012. CA Cancer J Clin.

[3] Yancik R (1997). Epidemiology of cancer in the elderly. Current status and projections for the future. Rays.

[4] Thurmes P, Call T, Slager S (2008). Comorbid conditions and survival in unselected, newly diagnosed patients with chronic lymphocytic leukemia. Leuk Lymphoma.

[5] Hallek M, Fischer K, Fingerle-Rowson G (2010). Addition of rituximab to fludarabine and cyclophosphamide in patients with chronic lymphocytic leukaemia: a randomised, open-label, phase 3 trial. Lancet.

[6] Gribben JG (2010). Chronic lymphocytic leukemia: planning for an aging population. Expert Rev Anticancer Ther.

[7] Foon KA, Boyiadzis M, Land SR (2009). Chemoimmunotherapy with low-dose fludarabine and cyclophosphamide and high dose rituximab in previously untreated patients with chronic lymphocytic leukemia. J Clin Oncol.

[8] Smolej L (2012). Therapy of elderly/comorbid patients with chronic lymphocytic leukemia. Curr Pharm Des.

[9] Leporrier M (2004). Role of fludarabine as monotherapy in the treatment of chronic lymphocytic leukemia. Hematol J.

[10] Warren JL, Klabunde CN, Schrag D et al (2002) Overview of the SEER–Medicare data: content, research applications, and generalizability to the United States elderly population. Med Care 40(8 Suppl):IV-3–IV-18 10.1097/01.MLR.0000020942.47004.03 12187163

[11] Potosky AL, Riley GF, Lubitz JD (1993). Potential for cancer related health services research using a linked Medicare-tumor registry database. Med Care.

[12] Binet JL, Auquier A, Dighiero G (1981). A new prognostic classification of chronic lymphocytic leukemia derived from a multivariate survival analysis. Cancer.

[13] Rai KR, Sawitsky A, Cronkite EP (1975). Clinical staging of chronic lymphocytic leukemia. Blood.

[14] Danese MD, Griffiths RI, Gleeson M (2011). An observational study of outcomes after initial infused therapy in Medicare patients diagnosed with chronic lymphocytic leukemia. Blood.

[15] Warren JL, Harlan LC, Fahey A (2002). Utility of the SEER–Medicare data to identify chemotherapy use. Med Care.

[16] Klabunde CN, Legler JM, Warren JL (2007). A refined comorbidity measurement algorithm for claims-based studies of breast, prostate, colorectal, and lung cancer patients. Ann Epidemiol.

[17] Charlson ME, Pompei P, Ales KL (1987). A new method of classifying prognostic comorbidity in longitudinal studies: development and validation. J Chron Dis.

[18] Linn BS, Linn MW, Gurel L (1968). Cumulative illness rating scale. J Am Geriatr Soc.

[19] Parmelee PA, Thuras PD, Katz IR (1995). Validation of the Cumulative Illness Rating Scale in a geriatric residential population. J Am Geriatr Soc.

[20] de Groot V, Beckerman H, Lankhorst GJ (2003). How to measure comorbidity.A critical review of available methods. J Clin Epidemiol.

[21] Hines RB, Chatla C, Bumpers HL (2009). Predictive capacity of three comorbidity indices in estimating mortality after surgery for colon cancer. J Clin Oncol.

[22] Greenland S, Rothman K (1998). Modern epidemiology.

[23] Kurth T, Walker AM, Glynn RJ (2006). Results of multivariable logistic regression, propensity matching, propensity adjustment, and propensity-based weighting under conditions of nonuniform effect. Am J Epidemiol.

[24] National Comprehensive Cancer Network (2012) NCCN clinical practice guidelines in oncology: non-Hodgkin's lymphomas. Version 3.2012. Retrieved from: http://www.nccn.org/professionals/physician_gls/pdf/nhl.pdf. Accessed 4 Feb 2013

[25] Byrd JC, Rai K, Peterson BL (2005). Addition of rituximab to fludarabine may prolong progression-free survival and overall survival in patients with previously untreated chronic lymphocytic leukemia: an updated retrospective comparative analysis of CALGB 9712 and CALGB 9011. Blood.

[26] Eichhorst B, Goede V, Hallek M (2009). Treatment of elderly patients with chronic lymphocytic leukemia. Leuk Lymphoma.

[27] Goede V, Fischer K, Busch R et al (2014) Obinutuzumab plus chlorambucil in patients with CLL and coexisting conditions. N Engl J Med. doi:10.1056/NEJMoa1313984 10.1056/NEJMoa1313984 24401022

[28] Byrd JC, Murphy T, Howard RS (2001). Rituximab using a thrice weekly dosing schedule in B-cell chronic lymphocytic leukemia and small lymphocytic lymphoma demonstrates clinical activity and acceptable toxicity. J Clin Oncol.

[29] Huhn D, von Schilling C, Wilhelm M (2001). Rituximab therapy of patients with B-cell chronic lymphocytic leukemia. Blood.

[30] Wierda WG (2006). Current and investigational therapies for patients with CLL. Hematol Am Soc Hematol Educ Prog.

[31] Hallek M, Cheson BD, Catovsky D (2008). Guidelines for the diagnosis and treatment of chronic lymphocytic leukemia: a report from the International Workshop on Chronic Lymphocytic Leukemia updating the National Cancer Institute-Working Group 1996 guidelines. Blood.

[32] Lamanna N, Kalaycio M, Maslak P (2006). Pentostatin, cyclophosphamide, and rituximab is an active, well-tolerated regimen for patients with previously treated chronic lymphocytic leukemia. J Clin Oncol.

[33] Robak T, Smolewski P, Cebula B (2006). Rituximab combined with cladribine or with cladribine and cyclophosphamide in heavily pretreated patients with indolent lymphoproliferative disorders and mantle cell lymphoma. Cancer.

[34] Keating MJ, O'Brien S, Albitar M (2005). Early results of a chemoimmunotherapy regimen of fludarabine, cyclophosphamide, and rituximab as initial therapy for chronic lymphocytic leukemia. J Clin Oncol.

[35] Wang M, Burau KD, Fang S (2008). Ethnic variations in diagnosis, treatment, socioeconomic status, and survival in a large population-based cohort of elderly patients with non-Hodgkin lymphoma. Cancer.

[36] Shavers VL, Brown ML (2002). Racial and ethnic disparities in the receipt of cancer treatment. J Natl Cancer Inst.

[37] Lund JL, Sturmer T, Harlan LC et al (2013) Identifying specific chemotherapeutic agents in Medicare data: a validation study. Med Care 51(5):e27–34 10.1097/MLR.0b013e31823ab60f PMC3290707 22080337

[38] National Cancer Institute (2012). Number of Part D enrollees.

