# Supplementary figures and images for: Treatment practice in the elderly patient with chronic lymphocytic leukemia—analysis of the combined SEER and Medicare database
Source: Ann Hematol. 2014 Mar 18;93(8):1335–44. doi: 10.1007/s00277-014-2048-6 (PMC4082137; doi:10.1007/s00277-014-2048-6)

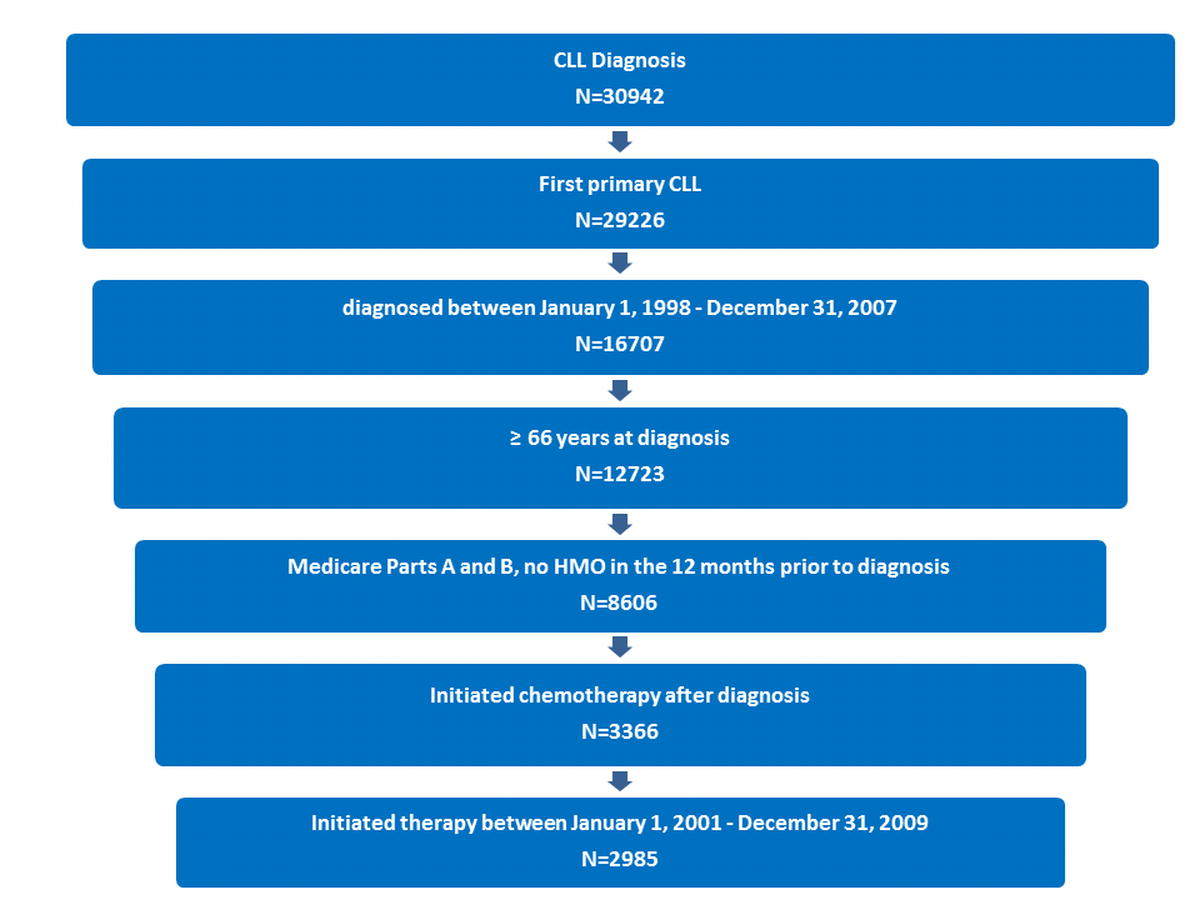

Supplement: Supplementary file 1 — (JPEG 80.1 kb) [file 277_2014_2048_Fig4_ESM.jpg]

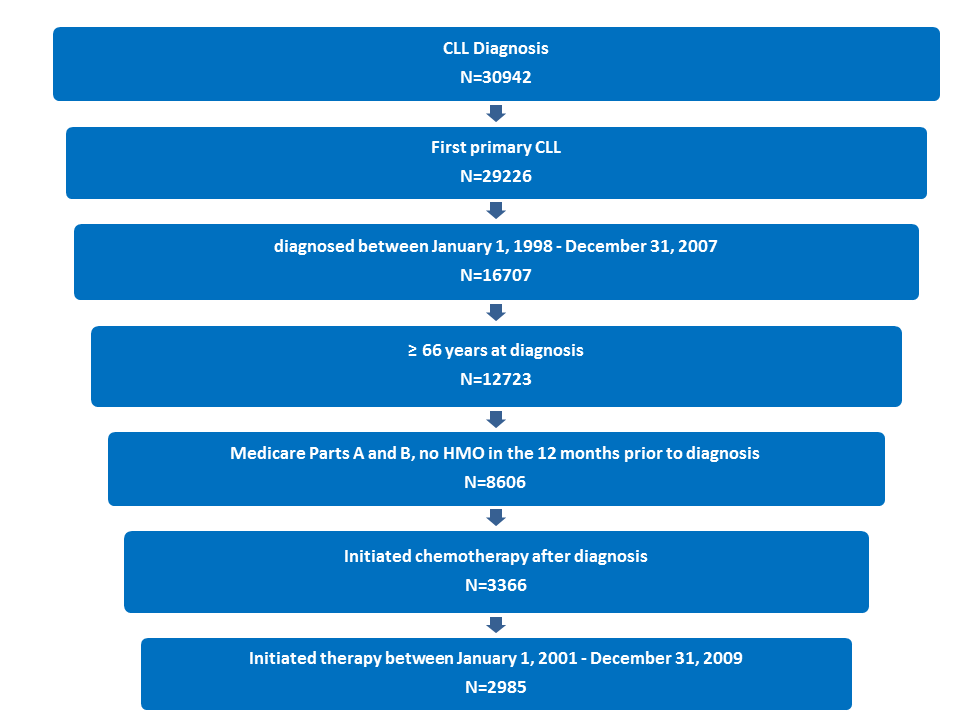

Supplement: Supplementary file 2 — (JPEG 39.5 kb) [file 277_2014_2048_MOESM1_ESM.tif]
